# Efficacy and safety of non-invasive ventilation in the treatment of acute cardiogenic pulmonary edema – a systematic review and meta-analysis

**DOI:** 10.1186/cc4905

**Published:** 2006-04-28

**Authors:** João C Winck, Luís F Azevedo, Altamiro Costa-Pereira, Massimo Antonelli, Jeremy C Wyatt

**Affiliations:** 1 Department of Pulmonology, Faculty of Medicine, University of Porto, Portugal; 2 Department of Biostatistics and Medical Informatics, Faculty of Medicine, University of Porto, Portugal; 3 Centre for Research in Health Technologies and Information Systems – CINTESIS (Centro de Investigação em Tecnologias e Sistemas de Informação em Saúde), Faculty of Medicine, University of Porto, Portugal; 4 Unita Operativa di Rianimazione e Terapia Intensiva, Instituto di Anestesia e Rianimazione, Policlinico Universitario A Gemelli, Universita Cattolica del Sacro Cuore, Rome, Italy; 5 Health Informatics Centre, University of Dundee, Dundee, Scotland, UK

## Abstract

**Introduction:**

Continuous positive airway pressure ventilation (CPAP) and non-invasive positive pressure ventilation (NPPV) are accepted treatments in acute cardiogenic pulmonary edema (ACPE). However, it remains unclear whether NPPV is better than CPAP in reducing the need for endotracheal intubation (NETI) rates, mortality and other adverse events. Our aim was to review the evidence about the efficacy and safety of these two methods in ACPE management.

**Methods:**

We conducted a systematic review and meta-analysis of randomized controlled trials on the effect of CPAP and/or NIPV in the treatment of ACPE, considering the outcomes NETI, mortality and incidence of acute myocardial infarction (AMI). We searched six electronic databases up to May 2005 without language restrictions, reviewed references of relevant articles, hand searched conference proceedings and contacted experts.

**Results:**

Of 790 articles identified, 17 were included. In a pooled analysis, 10 studies of CPAP compared to standard medical therapy (SMT) showed a significant 22% absolute risk reduction (ARR) in NETI (95% confidence interval (CI), -34% to -10%) and 13% in mortality (95%CI, -22% to -5%). Six studies of NPPV compared to SMT showed an 18% ARR in NETI (95%CI, -32% to -4%) and 7% in mortality (95%CI, -14% to 0%). Seven studies of NPPV compared to CPAP showed a non-significant 3% ARR in NETI (95%CI, -4% to 9%) and 2% in mortality (95%CI, -6% to 10%). None of these methods increased AMI risk. In a subgroup analysis, NPPV did not lead to better outcomes than CPAP in studies including more hypercapnic patients.

**Conclusion:**

Robust evidence now supports the use of CPAP and NPPV in ACPE. Both techniques decrease NETI and mortality compared to SMT and none shows increased AMI risk. CPAP should be considered a first line intervention as NPPV did not show a better efficacy, even in patients with more severe conditions, and CPAP is cheaper and easier to implement in clinical practice.

## Introduction

The public health burden of heart failure is very high. In the United States, heart failure is the most frequent cause of hospitalization in persons over 65 years of age [1], and in 2004, the estimated direct and indirect costs were 25.8 billion dollars [2]. A 4% hospital mortality due to heart failure was recently reported [3]. This rate increases to 36% in severe cases needing mechanical ventilation [4].

During the past 10 years, continuous positive airway pressure (CPAP) and non-invasive positive pressure ventilation (NPPV) have gained decisive roles in the management of various forms of respiratory failure [5][6]. Non-invasive ventilation achieves physiological improvement and efficacy similar to invasive ventilation [7], and by avoiding endotracheal intubation (ETI) reduces morbidity and complications [6].

Both NPPV and CPAP have been successfully used in patients with acute cardiogenic pulmonary edema (ACPE) [8,9]. A meta-analysis pooling data from three randomized controlled trials (RCTs) [10], published seven years ago, supported the efficacy of CPAP in avoiding ETI in ACPE patients, but showed no evidence of improved survival. Since that publication, several new RCTs have been published comparing NPPV, CPAP and standard medical therapy (SMT) in ACPE patients [11-25]. However, because most of them were small, several issues remain unresolved. The evidence about the size and significance of a reduction in mortality and about whether one technique is superior to the other remains unclear. Clinically important questions about which technique would lead to better outcomes in more hypercapnic patients [19] and about the best level of pressure support in NPPV [26] have also been raised, and may be preventing the wider use of these technologies.

Concerns have also been raised about safety issues related to non-invasive ventilation. Mehta and colleagues [25] showed, in an interim analysis of an RCT, an increased risk of acute myocardial infarction (AMI) in patients treated with NPPV. Due to the limited number of patients enrolled, however, those results were not conclusive, suggesting the need for a critical analysis of the safety of NPPV and CPAP in the treatment of ACPE.

A very recent meta-analysis unfortunately addressed only some of the questions to which clinicians need answers. Masip and colleagues [27], showed that non-invasive ventilation – jointly considering CPAP and NPPV together as if they were the same technology – was associated with a 43% relative risk reduction in mortality and 56% relative risk reduction in the need for ETI, and found no significant differences in efficacy between those two modalities. An important criticism of this review is that it presents results for non-invasive ventilation (pooling CPAP and NPPV together) and consequently double counting control group patients in three studies (with three arms), inflating the number of patients included and having potential impact on the calculated confidence intervals and conclusions. Moreover, this meta-analysis failed to include two useful studies (one inappropriately excluded and one not found). It also did not analyze evidence about differences in efficacy in the subset of more hypercapnic patients or about differences related to the level of pressure support in NPPV. It commented on but did not present relevant data, or thoroughly analyze, the potentially increased AMI risk associated with non-invasive ventilation, another issue that concerns clinicians. Finally, the results of this meta-analysis were presented using the relative risk scale, which is less easy to translate to practice and more challenging for clinicians to understand.

The aim of our study was to systematically review the evidence in order to answer key clinical questions about the efficacy and safety of CPAP and NPPV in the treatment of patients with ACPE, considering three different outcomes: the need for ETI; in-hospital all cause mortality; and incidence of newly developed AMI. We specifically and separately addressed three different comparisons: CPAP and SMT versus SMT alone; NPPV and SMT versus SMT alone; and NPPV and SMT versus CPAP and SMT. Secondary aims were to analyze the impact of patients' baseline hypercapnia on the efficacy of CPAP and NPPV and to test a common clinical hypothesis about the advantage of NPPV when using higher levels of pressure support ventilation.

## Materials and methods

### Study design

A systematic review and meta-analysis of RCTs focusing on the effect of CPAP and NPPV in the treatment of ACPE was undertaken. The methodological approach included the development of selection criteria, definition of search strategies, quality assessment of the studies, data abstraction and statistical data analysis [28].

### Selection criteria

The study selection criteria were defined before data collection, in order to properly identify high quality studies eligible for the analysis.

The following inclusion criteria were defined. Patient population: adult patients presenting to hospital with ACPE, defined as existence of dyspnea of sudden onset, increased respiratory rate, a compatible physical examination (bilateral crackles on pulmonary auscultation, elevated jugular venous pressure, third heart sound on cardiac auscultation), bilateral pulmonary infiltrates on chest radiograph plus significant hypoxemia. Study design: prospective randomized parallel trials with independent randomization of ACPE patients. Interventions: use of CPAP (delivered using any device) and medical therapy compared with standard medical therapy alone; use of NPPV (with any device) and medical therapy compared with standard medical therapy alone; or use of CPAP and medical therapy compared with NPPV and medical therapy. Outcomes: need for ETI as decided by trialists, all-cause mortality and risk of newly developed AMI after delivery of study interventions.

To improve the internal validity of this meta-analysis, we decided to consider separately trials of NPPV and CPAP, because these two methods have different technical, physiological and clinical characteristics. Pooling those two interventions in a single 'non-invasive ventilation' intervention may not be appropriate and could have led to additional heterogeneity and patient overlap in trials with three arms. Also, trials that included both acute respiratory failure and ACPE patients [29-33] were included only if there was independent stratified randomization of therapies for this sub-group.

### Search strategy

Our primary method to locate potentially eligible studies was a computerized literature search in the MEDLINE database, from inception to May 2005, without any restriction on language of publication, using the following search keywords and MeSH terms: (artificial respiration or continuous positive airway pressure or non-invasive positive pressure ventilation or non-invasive ventilation or non-invasive ventilation) and (pulmonary edema or pulmonary oedema or congestive heart failure) and (clinical and trial or clinical trials or clinical trial or random* or random allocation or therapeutic use). Literature searches were also undertaken, using the same search keywords, in the following databases: the American College of Physicians (ACP) Journal Club Database; the Cochrane Central Register of Controlled Trials (CCTR); the Cochrane Database of Systematic Reviews (CDSR); the Digital Academic Repositories (DARE) Database; and the MetaRegister of Controlled Trials at Current Controlled Trials webpage.

In defining all search strategies we gave priority to formats with higher sensitivity, in order to increase the probability of identifying all relevant articles.

We also reviewed the references of all relevant articles and review articles, hand searched abstracts and conference proceedings of recent relevant congresses and scientific forums from 2000 to 2005, and contacted authors and experts working in this field.

### Study quality assessment and data abstraction

In the first phase of selection, the titles and abstracts of the retrieved studies were screened for relevance by two reviewers. In the second phase, two reviewers (ALF and WJC) independently analyzed the full-papers of articles identified as potentially relevant. Selection criteria were applied, exclusions were decided and disagreements settled by consensus. Data abstraction for quality assessment and pooled analysis was performed independently using a previously specified standardized form. Quality assessment considered two types of study quality criteria, general and specific.

The general quality criteria included methodological and reporting characteristics of RCTs generally accepted as appropriate to evaluate this type of study (Table 1). The specific quality criteria included characteristics specifically relevant to RCTs studying ACPE patients and the effect of non-invasive ventilation (Table 1).

### Statistical analysis

For the pooled assessment of treatment effects in the three comparisons (CPAP versus SMT, NPPV versus SMT and CPAP versus NPPV) and the three outcome variables (need for ETI, mortality and AMI risk) in this review, we used the Mantel-Haenszel method for fixed effects estimation and the DerSimonian and Laird method for random effects estimation. One problem that could have arisen in the pooled analysis is that of patient overlap because of the inclusion of studies with three arms (CPAP, NPPV and SMT)[15,21,23]. To overcome this problem, among other previously stated reasons, we separately considered the three comparisons CPAP versus SMT, NPPV versus SMT and CPAP versus NPPV.

We used risk difference (absolute risk reduction) as the scale for measuring efficacy and side effects because clinicians find it a more intuitive and interpretable metric as it measures the absolute difference between outcome risks in intervention and control groups, rather than odds ratios or relative risks, which many clinicians and patients find hard to understand [34,35].

Heterogeneity of treatment effects was assessed by graphical inspection of forest plots and formally using the Q statistic (at a *p *value ≤ 0.1) and I^2 ^statistic for estimating inconsistency among study results. The random effects model for pooling effects was preferred and always used if heterogeneity of treatment effects was present. Subgroup and sensitivity analysis were performed following a predefined protocol and considering the hypothesis previously presented.

Potential publication bias was assessed by visual analysis of the funnel plots, which allows evaluation of publication bias by presenting the study's risk difference plotted as a function of its standard error, and then formally checked by the rank correlation test of Begg [36].

The data processing and statistical analysis were performed using the Cochrane Collaboration's Review Manager Software version 4.2 [37] and RevMan Analyses software version 1.0 [38].

## Results

### Search and study selection

**Figure 1:**
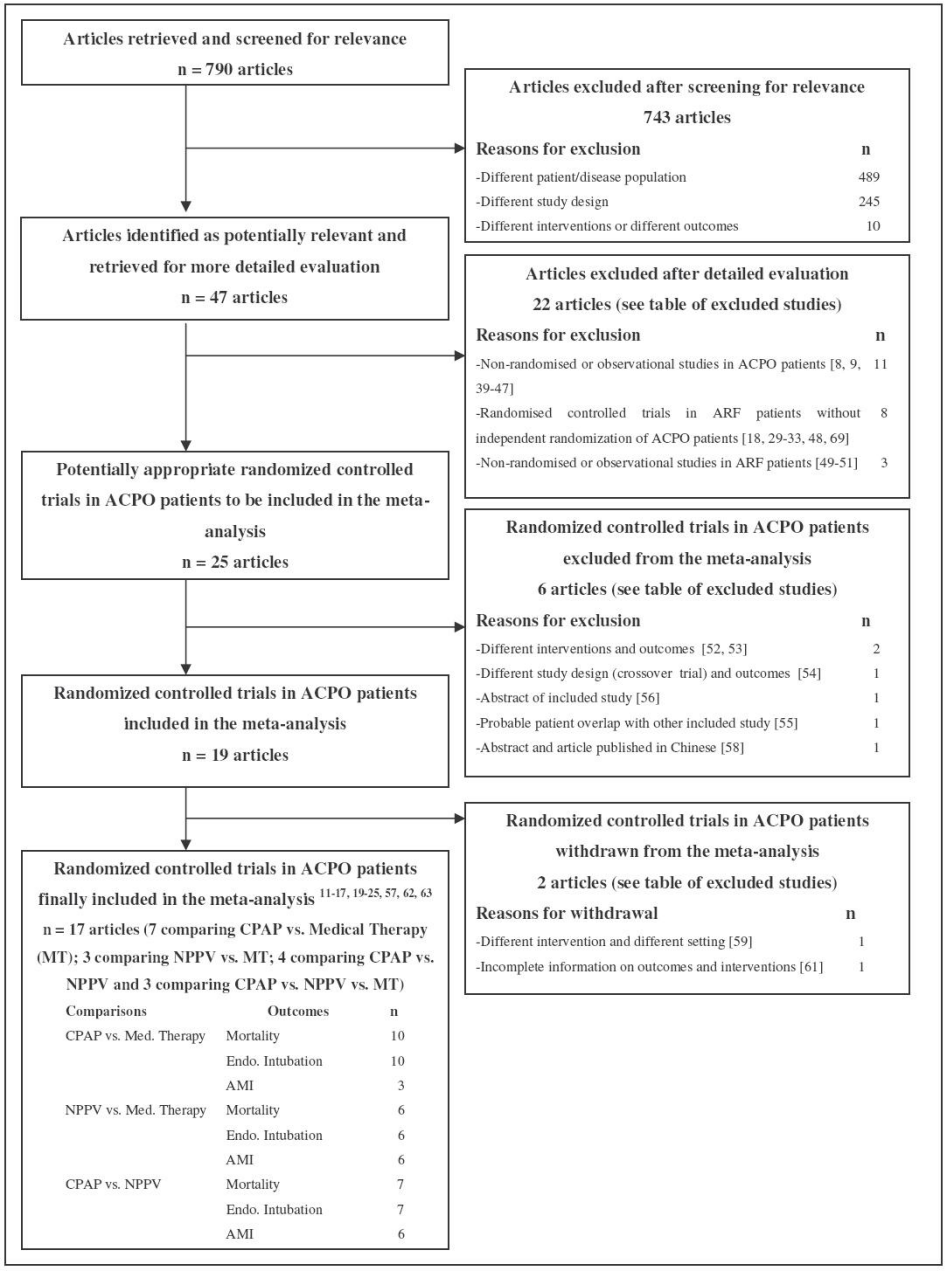
Flow chart of the study selection process. ACPO, acute cardiogenic pulmonary edema; ARF = acute respiratory failure; CPAP, continuous positive airway pressure ventilation; ETI, endotracheal intubation; NPPV, non-invasive positive pressure ventilation; MT, medical therapy.

A total of 790 articles were identified using the search strategy and sources listed. After screening titles and abstracts for relevance, 744 articles were excluded (the reasons for exclusion are presented in Figure 1). The remaining 46 articles were retrieved for more detailed full paper evaluation and 22 were excluded [18,39-51] (Figure 1). Eight other articles reporting randomized controlled trials in ACPE patients were excluded, for the following reasons: two because different interventions were studied [52,53]; one because it was a randomized cross-over trial focusing on physiological outcome variables [54]; two because of probable patient overlap [55,56] with the included studies by Crane and colleagues [21] and Lin and colleagues [57]; one because its abstract and full paper were published in Chinese [58]; one (by Sharon and colleagues [59]) because it was performed in a pre-hospital setting and had a different intervention in the control group – SMT plus high dose IV isossorbide-dinitrate – and has been frequently criticized for its methodological problems [60]; and one conference abstract by Liesching and colleagues reporting an RCT comparing NPPV versus CPAP was withdrawn because it was not possible to obtain the minimum information on study design, patients, interventions and outcomes [61].

Unlike the other meta-analysis previously published [27], we did not excluded the article by Takeda and colleagues [12] because there is no evidence of patient overlap with the other study by the same authors [11]. Patient inclusions in the two articles have different time frames and settings [11,12].

The final study cohort consisted of 17 studies: seven comparing CPAP with SMT [11,12,16,22,57,62,63], three comparing NPPV with SMT [13,14,19], four comparing CPAP directly with NPPV [17,20,24,25] and three studies each with three arms comparing CPAP, NPPV and SMT [15,21,23] (Table 2).

### Methodological quality of included studies

Study quality assessment considered two types of criteria: general and specific. The general quality criteria are presented in Table 2. The studies had generally small sample sizes (median, 40 patients; range, 22 to 130); the total number of patients included was 938. Most of them had adequate randomization concealment and adequate selection criteria. Four out of 17 did not report an intention-to-treat analysis. Only one study blinded physicians, nurses and patients to the intervention by covering the control panel of the ventilator. Almost none of the studies reported or commented on blinding strategies. Most of them reported on strategies for standardization of co-interventions and had complete follow-up details for all participants. Six out of 17 studies had inadequate or unclear outcome definitions.

The specific quality criteria are presented in Table 3. There were several different definitions of ACPE (see Table 3), but most of them included the basic criteria considered in our definition (existence of dyspnea of sudden onset, increased respiratory rate, a compatible physical examination, bilateral crackles on pulmonary auscultation, elevated jugular venous pressure, third heart sound on cardiac auscultation, bilateral pulmonary infiltrates on chest radiograph plus significant hypoxemia). Inclusion and exclusion criteria had some variability (Table 3), with some studies including much selected groups of patients. Baseline co-morbidities differed between studies and in some studies the presence of chronic obstructive pulmonary disease (COPD) or AMI was considered as exclusion criteria (Table 3). The frequency of AMI at baseline, for each study, is presented in Table 3. Major differences were found among studies regarding the methods of implementation and technical characteristics of the ventilation devices (Table 3) and regarding the definition and adequacy of criteria for ETI (Table 3).

The analysis of safety issues will mainly focus on comparisons of AMI risk among interventions. Some other adverse events of non-invasive ventilation were reported sporadically by authors (facial erythema, nasal skin necrosis, vomiting, gastric distension, pulmonary aspiration, barotrauma and asphyxia), but were always described as rare events.

### Continuous positive airway pressure ventilation versus standard medical therapy

**Figure 2:**
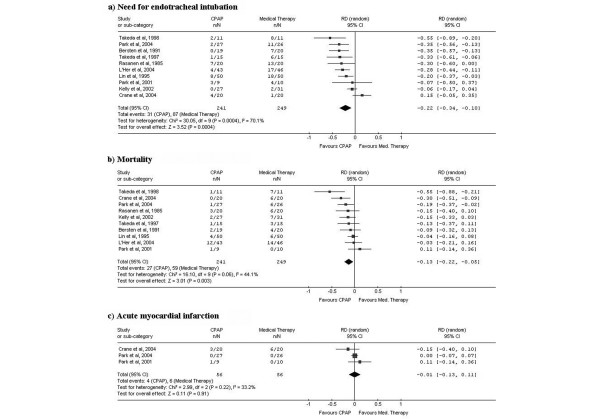
Results and pooled analysis of absolute risk differences (RDs) for the outcomes **(a) **need for endotracheal intubation, **(b) **mortality and **(c) **acute myocardial infarction in trials comparing continuous positive airway pressure ventilation (CPAP) versus medical therapy in acute cardiogenic pulmonary edema patients.

Results of studies comparing CPAP therapy with SMT are presented in Figure 2. In the random effects pooled analysis, CPAP therapy showed a statistically significant 22% risk reduction in need for ETI (95% confidence interval (CI), -34% to -10%; *p* = 0.0004) and a 13% risk reduction for mortality (95%CI, -22% to -5%; *p* = 0.0003). Significant heterogeneity was found in the pooled analysis of need for ETI and borderline significant heterogeneity was found for mortality (Cochran's Q chi-square test, *p* = 0.0004; I^2 ^= 70.1% for intubation and Cochran's Q chi-square test, *p* = 0.060; I^2 ^= 44.1% for mortality). Nevertheless, all studies but one found a reduction of risk in the CPAP group. Heterogeneity is in part related to the extreme findings of 55% risk reduction for both ETI and mortality in the study by Takeda and colleagues [12].

Only three studies included data on myocardial infarction and the random effects pooled analysis showed no difference in AMI risk between the CPAP and SMT groups (Risk Difference – RD, -1%; 95%CI, -13% to 11%; *p *= 0.910) and non-significant heterogeneity (Cochran's Q chi-square test, *p *= 0.22; I^2 ^= 33.2%).

### Non-invasive positive pressure ventilation versus standard medical therapy

**Figure 3:**
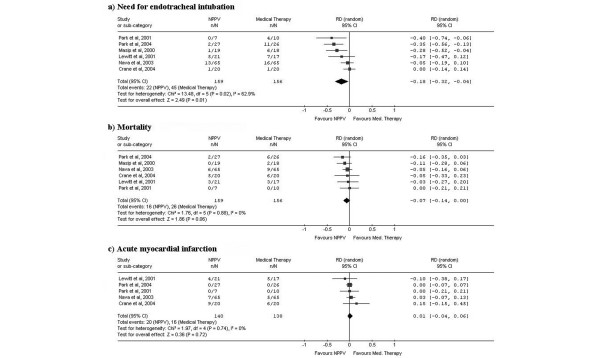
Results and pooled analysis of absolute risk differences (RDs) for the outcomes **(a) **need for endotracheal intubation, **(b) **mortality and **(c) **acute myocardial infarction in trials comparing non-invasive positive pressure ventilation (NPPV) versus medical therapy in acute cardiogenic pulmonary edema patients.

Results of the studies comparing NPPV with SMT are presented in Figure 3. The random effects pooled analysis showed a statistically significant 18% risk reduction in need for ETI (95%CI, -32% to -4%; *p *= 0.010) and a non-significant 7% risk reduction for mortality (95%CI, -14% to 0%; *p *= 0.060) favoring the NPPV group. Significant heterogeneity was found in the pooled analysis of need for ETI (Cochran's Q chi-square test, *p *= 0.02; I^2 ^= 62.9%), but again, all studies but one showed risk reduction for the NPPV group.

Random effects pooled analysis of risk differences for AMI showed a small but non-significant risk increase for the NPPV group (RD, 1%; 95%CI, -4% to 5%; *p *= 0.720).

To test the clinical hypothesis about an advantage of NPPV over SMT when using higher levels of pressure support ventilation [26], we performed a predefined subgroup analysis (Forest plots not presented but available on request) with stratification based on the level of pressure support ventilation (Pressure Support Ventilation – PSV ≥ 10.0 cmH2O versus PSV < 10.0 cmH2O; Table 3). In the subgroup of studies with higher levels of pressure support ventilation [13,21], a random effects pooled analysis showed a statistically non-significant risk reduction in need for ETI (RD, -13%; 95%CI -44% to 19%; *p *= 0.430; Cochran's Q chi-square test for heterogeneity, *p *= 0.020) and mortality (RD, -9%; 95%CI -24% to 5%; *p *= 0.190; Cochran's Q chi-square test for heterogeneity, *p *= 0.670) favoring the NPPV group. In the subgroup of studies with lower levels of pressure support ventilation [14,15,19,23], the random effects pooled analysis showed a statistically significant risk reduction in need for ETI (RD, -22%; 95%CI -40% to -3%; *p *= 0.020; Cochran's Q chi-square test for heterogeneity, *p *= 0.060) and a non-significant risk reduction for mortality (RD, -6%; 95%CI -14% to 2%; *p* = 0.160; Cochran's Q chi-square test for heterogeneity, *p *= 0.690) favoring the NPPV group.

### Continuous positive airway pressure ventilation versus non-invasive positive pressure ventilation

**Figure 4:**
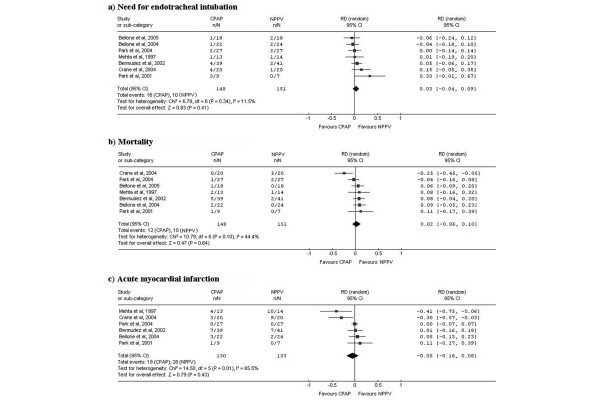
Results and pooled analysis of absolute risk differences (RDs) for the outcomes **(a) **need for endotracheal intubation, **(b) **mortality and **(c) **acute myocardial infarction in trials comparing of continuous positive airway pressure ventilation (CPAP) versus non-invasive positive pressure ventilation (NPPV) in acute cardiogenic pulmonary edema patients patients.

Results from studies directly comparing CPAP with NPPV are presented in Figure 4. The random effects pooled analysis showed a statistically non-significant need for ETI risk reduction (RD, 3%; 95%CI -4% to 9%; *p* = 0.041) and mortality reduction (RD, 2%; 95%CI -6% to 10%; *p* = 0.640) in the NPPV group. No evidence of significant heterogeneity in need for ETI was found (Cochran's Q chi-square test, *p* = 0.340; I^2 ^= 11.5%). Heterogeneity with borderline significance was found for mortality (Cochran's Q chi-square test, *p* = 0.100; I^2 ^= 44.4%). A fixed effects pooled analysis, which could be considered appropriate in this case due to the absence of heterogeneity, obtained similar non-significant results (RD, 4%; 95%CI -2% to 10% and RD, 2%; 95%CI -5% to 8% for ETI and mortality, respectively).

Random effects pooled analysis of risk differences for AMI showed a non-significant risk reduction in the CPAP group (RD, -5%; 95%CI, -18% to 8%; *p *= 0.430).

**Figure 5:**
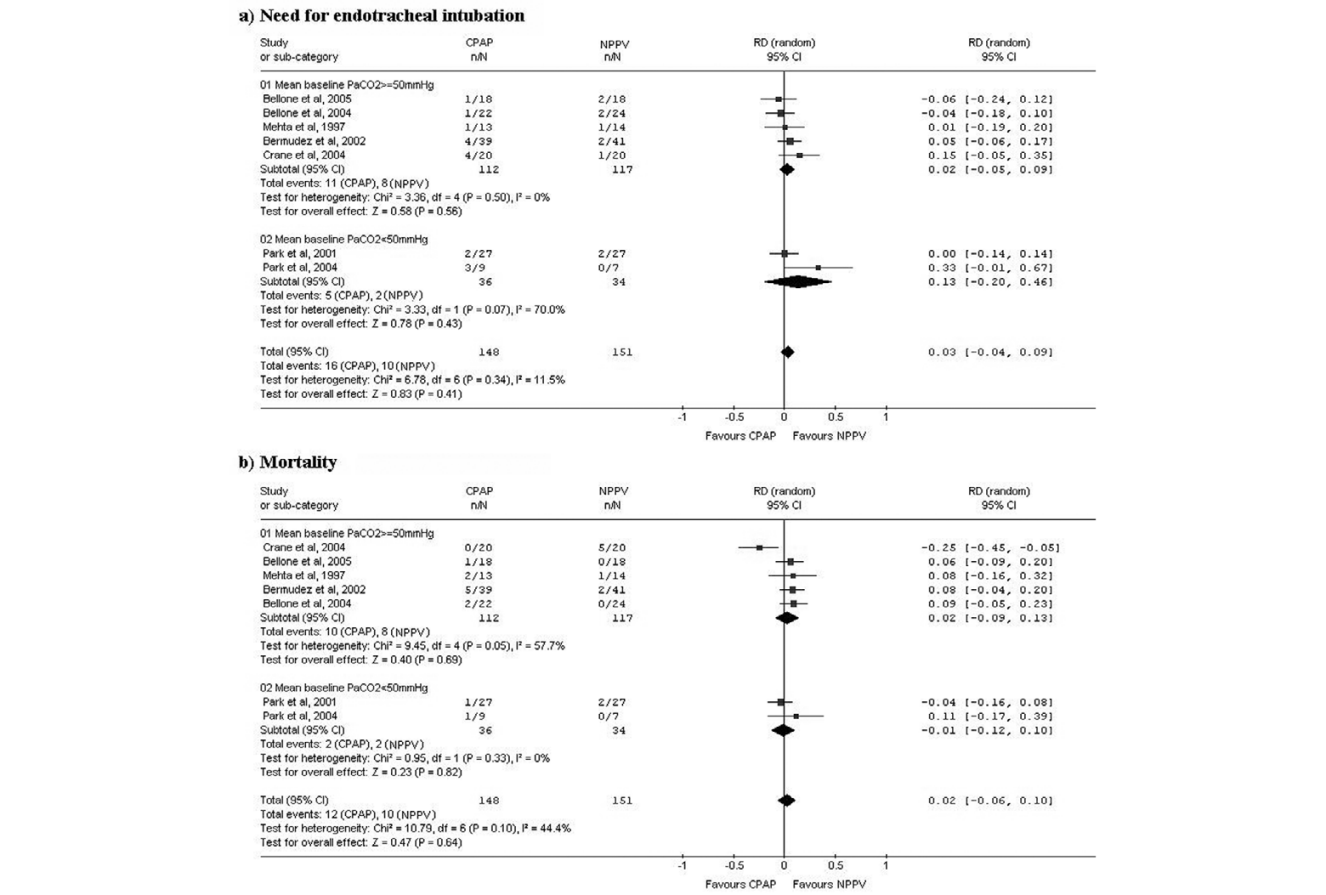
Results and pooled analysis of absolute risk differences (RDs) for the outcomes **(a) **need for endotracheal intubation, **(b) **mortality and **(c) **acute myocardial infarction in trials comparing of continuous positive airway pressure ventilation (CPAP) versus non-invasive positive pressure ventilation (NPPV) in acute cardiogenic pulmonary edema patients patients. Subgroup analysis with stratification by baseline PaCO_2 _level.

To explore the hypothesis proposed by some clinicians on the advantage of NPPV over CPAP in hypercapnic patients [19], we analyzed the impact of patients' baseline hypercapnia in the comparison between CPAP and NPPV. A subgroup analysis was performed (Figure 5) with stratification based on mean baseline level of arterial carbon dioxide pressure, (PaCO_2 _<50 mmHg versus PaCO_2 _≥ 50 mmHg). In the group of studies with more hypercapnic patients at baseline, the random effects pooled analysis showed a statistically non-significant risk reduction in need for ETI (RD, 2%; 95%CI -5% to 9%; *p *= 0.560) and mortality (RD, 2%; 95%CI -9% to 13%; *p *= 0.690) favoring the NPPV group. In the group of studies with less hypercapnic patients at baseline, the random effects pooled analysis showed a statistically non-significant risk reduction in need for ETI (RD, 13%; 95%CI -20% to 46%; *p *= 0.430) and a non-significant risk increase for mortality (RD, -1%; 95%CI -12% to 10%; *p *= 0.820) for the NPPV group.

### Publication bias

**Figure 6:**
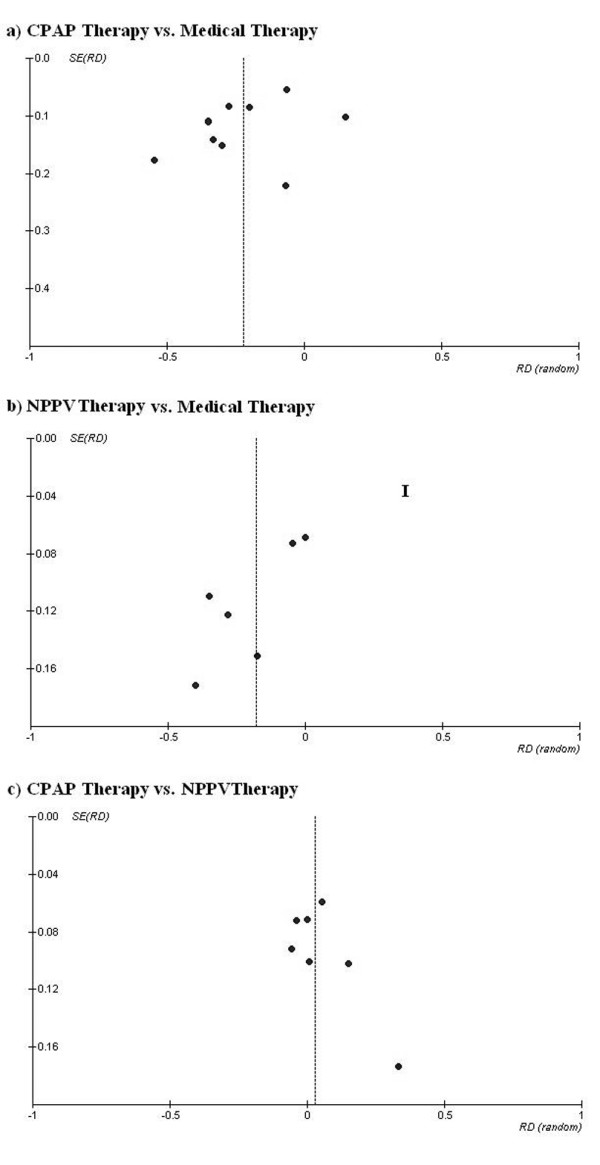
Funnel plots with effect measures (risk difference (RD)) as a function of its standard error (SE) for the outcome endotracheal intubation in trials comparing **(a) **continuous positive airway pressure ventilation (CPAP) versus medical therapy; **(b) **non-invasive positive pressure ventilation (NPPV) versus medical therapy and CPAP versus NPPV.

Funnel plots are presented in Figure 6. Although separate analyses for all outcomes and comparisons were performed, we only present here the analysis of potential publication bias for the need for ETI, because results regarding other outcomes are very similar.

For the comparison of CPAP versus SMT, the funnel plot is approximately symmetrical, but larger studies (more precise measures of effect) tend to have smaller effects and smaller studies (less precise measures of effect) tend to have larger effects. The rank correlation test of Begg gives a non-significant result (*p *= 0.325), so the absence of publication bias cannot be rejected. For the comparison of NPPV versus SMT the funnel plot is asymmetrical and seems to indicate a lack of small studies with small effects. The rank correlation test of Begg gives a non-significant result (*p *= 0.251). For the comparison of CPAP versus NPPV the funnel plot is asymmetrical and indicates a lack of small studies with effects favoring CPAP therapy. For this comparison, there seems to be some evidence of a publication bias, favoring the publication of studies with positive results for NPPV therapy. Nonetheless, the rank correlation test of Begg gives, once again, a non-significant result (*p *= 0.129).

## Discussion

ACPE is a rather common condition and may require mechanical ventilation [64], leading to high in-hospital mortality. The use of non-invasive ventilation to treat ACPE was first described by Poulton and colleagues [65] more than 60 years ago, and seven years ago the first meta-analysis appeared, showing the efficacy of CPAP in the treatment of ACPE [10]. Since then, several RCTs comparing the use of CPAP and NPPV with SMT or with each other have been published, and the role of non-invasive ventilation, and specially CPAP, in ACPE patients is becoming more clearly defined.

The present meta-analysis focused on important, unresolved clinical questions about the efficacy and safety of these techniques that could be delaying their uptake in most centers.

First, the meta-analysis shows that, in patients with ACPE, CPAP and NPPV, both significantly decrease need for ETI risk, and CPAP alone significantly reduces mortality when compared to SMT. Both NPPV and CPAP appear to be equivalent in reducing need for ETI and mortality. NPPV does not yet show a significant reduction in mortality, probably due to the low power related to the limited number of patients in the studies analyzed.

To put this evidence of clinical efficacy into context, it is also important to take into account the costs and difficulties involved in implementing non-invasive ventilation in clinical practice and the logistic differences between using the two non-invasive ventilation techniques. It is clear that CPAP is more easily implemented in clinical practice and that it carries smaller associated costs [53]. In fact, the cost-effectiveness of CPAP has already been demonstrated [66].

Second, our analysis of the safety of these methods showed that, although some caution is still advised, there is no evidence of increased risk of AMI with either of these techniques and the other adverse events described with these techniques are very rare. Although one study [25] found a higher incidence of AMI with NPPV, subsequent research has not confirmed this finding [13,19-21,23]. In the present meta-analysis, there was no significant difference in the risk of AMI between CPAP and NPPV when compared to SMT. Careful and frequent monitoring of patients with ACPE is mandatory, especially in the presence of AMI, but there is no evidence from these trials to contraindicate the use of NPPV.

Third, in a subgroup analysis of studies including patients with mean baseline PaCO_2 _levels below and above 50 mmHg, NPPV showed only a small trend towards decreased need for ETI and mortality, so the suggested superiority of NPPV in hypercapnic ACPE patients due to respiratory muscle unloading [19] was not confirmed. Although a number of studies in our meta-analysis included patients with ACPE and coexisting COPD [13,19-21], who one would expect to benefit the most from NPPV [67], studies including hypercapnic ACPE patients without COPD also showed significant improvement in PaCO_2 _with CPAP [24].

Fourth, in a subgroup analysis we found no evidence supporting the clinical hypothesis about the advantage of NPPV over SMT when using higher levels of pressure support ventilation [26,13,21].

Although our conclusions appear robust and well supported by the evidence, this meta-analysis has some limitations that should be pointed out. We found important clinical differences among the studies included in the analysis. The patients selected may not be completely comparable from study to study. Specifically, we found relevant differences relating to the etiology of ACPE. The mortality rate in the control groups had a wide range (from 0% [15] to 64% [12]), indicating large differences in severity of illness between studies. In addition, the rates of AMI on admission, one of the most important predictors of mortality [4,64,68] varied from 0% in Bellone and colleagues [20] to 100% in Takeda and colleagues' study [12].

Some of the studies included had moderate methodological limitations. When analyzing the comparison between NPPV and SMT, some concern may be raised about study recruitment and randomization procedures. In fact, one study had significantly more patients with a history of AMI, COPD and diabetes mellitus and patients with higher baseline PaCO_2 _levels randomized to the control group [13]. Studies comparing CPAP with NPPV also had problems with baseline differences between groups. For instance, in Park and colleagues' study [15], patients treated with CPAP were more severely ill than those treated with NPPV, and in the study by Crane and colleagues [21], the NPPV group had significantly more co-morbidities, lower PaCO_2 _and a trend toward higher median peak creatine kinase (CK) levels. These differences could potentially account for the advantage of NPPV in reducing ETI in Park and colleagues' study [15] and for the advantage of CPAP in reducing mortality in Crane and colleagues' study [21].

Important heterogeneity was also found in relation with outcome definitions. The criteria and time frame used in the definition of patients needing ETI was very different from study to study. Moreover, some studies considered the need for intubation as the outcome whereas others considered actual intubation. The creation of consensus guidelines for outcome definitions for this type of study would be very useful to promote further rigorous research and would support future systematic reviewers.

Differences were also found in the technical specifications of the ventilation devices studied. Although face mask was the main interface used, in some studies [11,12,14,25] a nasal mask or a combination of the two were applied. Duration of non-invasive ventilation and the type of ventilator may have also influenced outcomes, and this was not examined in this review. Some different kinds of interfaces and ventilatory modes have produced better patient comfort [69,70], but they do not seem to have a major impact on survival or other outcomes. The differences relating to ventilators and interfaces among studies included in this meta-analysis do not seem to account for the differences in the results.

Finally, a search for potential publication bias was performed using funnel plots and the rank correlation test of Begg. Using these methods, it is not possible to rule out the hypothesis of publication bias in our meta-analysis. We found some evidence indicating that smaller studies are more likely to be published if they have larger effects and some evidence of a publication bias favoring the publication of studies with positive results for NPPV therapy when compared to CPAP. We should remember, though, that the rank correlation test of Begg has low power. It is also important to emphasize that the asymmetry found in funnel plots could be related to several other sources of bias, and is not necessarily evidence of publication bias.

## Conclusion

The evidence for the advantage of non-invasive ventilation techniques, and especially of CPAP, over SMT is now robust, and its use as a first line intervention in ACPE patients is becoming mandatory. Although one recent guideline for the treatment of ACPE suggests CPAP to avoid ETI and mechanical ventilation [71], this technique is still underused in many clinical centers, partly because the clinical questions we address in this meta analysis had not been answered. Although both techniques, CPAP and NPPV, showed similar efficacy in decreasing need for ETI and mortality without increasing the risk of AMI, from a practical point of view CPAP has been shown to be cheaper and easier to use and implement in clinical practice [53], so it could be considered the preferred intervention in ACPE patients.

Finally, we think it is important for researchers in this field to create consensus guidelines over methods for reporting and defining population, interventions and outcome measures. Taking into account the evidence presented here, it does not seem advisable, from an ethical point of view, to pursue further research comparing non-invasive ventilation methods with SMT in ACPE patients. Research in the future should concentrate on the definition of subgroups of patients for whom NPPV could eventually have advantage over CPAP, the optimal levels of pressure when using NPPV and definition of the best time to start non-invasive ventilation.

## Key messages

• 	CPAP and NPPV have gained decisive roles in the management of various forms of respiratory failure, namely ACPE.

• 	In this meta-analysis we show that, in ACPE patients, CPAP and NPPV both significantly decrease the need for ETI, and CPAP significantly reduces mortality when compared to SMT. The evidence is now robust, and the use of these techniques as a first line intervention in ACPE patients is becoming mandatory.

• 	Although both techniques, CPAP and NPPV, showed similar efficacy in decreasing need for ETI and mortality, CPAP has been shown to be cheaper and easier to use and implement in clinical practice, so it could be considered the preferred intervention in ACPE patients.

• 	Analysis of the safety of these methods showed that, although some caution is still advised, there is no evidence of increased risk of AMI with either of these techniques and other adverse events described are very rare.

• 	No evidence supporting the suggested superiority of NPPV in hypercapnic ACPE patients was found and the advantage of higher levels of pressure support ventilation when using NPPV was not confirmed.

## Abbreviations

ACPE = acute cardiogenic pulmonary edema; AMI = acute myocardial infarction; CI = confidence interval; COPD = chronic obstructive pulmonary disease; CPAP = continuous positive airway pressure ventilation; ETI = endotracheal intubation; NPPV = non-invasive positive pressure ventilation; RCT = randomized controlled trial; SMT = standard medical therapy.

## Competing interests

The authors declare that they have no conflicting interests.

## Authors' contributions

JCW is responsible for initiation and direction of the review. LFA is responsible for study design, methods and statistical analysis. JCW and LFA selected studies and extracted data. JCW, LFA and AC-P interpreted results and wrote the manuscript. JCW and MA critically reviewed the manuscript for important intellectual content.

## Figures and Tables

**Table 1:** General and specific quality criteria

**General quality criteria**

Sample size (total number of participants)
Randomization allocation concealment (adequate, inadequate or uncertain)
Objective selection criteria for participants:
Yes: if inclusion and exclusion criteria for participants are adequately reported
No: if selection criteria are not reported
Blinding:
Yes: for articles that implemented blinding at any level
No: for articles reporting not being able to implement blinding of interventions at any level
Not reported: for articles that did not make any mention of blinding
Standardization of co-interventions:
Yes: if there was an attempt to standardize treatment and care besides the assigned interventions
No: if no attempt to standardize was applied
Uncertain: if this was not clearly reported
Intention-to-treat analysis (adequate, inadequate or uncertain)
Complete follow-up details (yes, no, not reported)
Outcome definition:
Adequate: if objective criteria for endotracheal intubation were defined
Inadequate: if the criteria were not defined
Uncertain: if application of criteria was unclear
**Specific quality criteria**
Patient selection criteria (inclusion and exclusion)
Type of patients (presence of baseline co-morbidity: AMI or chronic obstructive pulmonary disease)
Description of baseline criteria for severity of illness
Report of interventions (technical description of CPAP and NPPV methods)
Report of objective criteria for endotracheal intubation (adequate, inadequate or uncertain)

CPAP, continuous positive airway pressure ventilation; NPPV, non-invasive positive pressure ventilation.

**Table 2:** General characteristics and general quality criteria of randomized trials in acute cardiogenic pulmonary edema patients included in the study

Reference	Country and Setting	Sample size	Interventions	Outcomes analyzed	Randomization assignment concealment^a^	Objective selection criteria^b^	Blinding^c^	Standardization of co-interventions^d^	Intention-to-treat analysis^e^	Complete follow-up details^f^	Outcome definition^g^
Rasanen *et al*. 1985 [62]	Finland: ED and ICU	40	SMT vs CPAP	Meeting criteria for ETI during 3 h follow-up; in-hospital mortality	Adequate	Yes	NR	Yes	Adequate	Yes	Adequate
Bersten *et al*. 1991 [63]	Australia: ICU	39	SMT vs CPAP	Meeting criteria for ETI during 24 h follow-up; in-hospital mortality	Uncertain	Yes	No	Yes	Uncertain	Yes	Adequate
Lin *et al*. 1995 [57]	Taiwan: ICU	100	SMT vs CPAP	Meeting criteria for ETI during 6 h follow-up; in-hospital mortality	Uncertain	Yes	NR	Yes	Adequate	Yes	Adequate
Takeda *et al*. 1997 [11]	Japan: CU	30	SMT vs CPAP	Meeting criteria for ETI during 24 h follow-up; in-hospital mortality	Uncertain	Yes	NR	Yes	Adequate	Yes	Adequate
Takeda *et al*. 1998 [12]	Japan: CU	22	SMT vs CPAP	Meeting criteria for ETI during 48 h follow-up; in-hospital mortality	Adequate	Yes	NR	Yes	Adequate	Yes	Adequate
Kelly *et al*. 2002 [16]	Scotland, UK: ED and HDU	58	SMT vs CPAP	Meeting criteria for treatment failure; in-hospital mortality	Adequate	Yes	NR	Yes	Adequate	Yes	Inadequate
L'Her *et al*. 2004 [22]	France: ED	89	SMT vs CPAP	Meeting criteria for ETI or death during 48 h follow-up; in-hospital mortality	Adequate	Yes	NR	Yes	Adequate	Yes	Adequate
Masip *et al*. 2000 [13]	Spain: ED and ICU	37	SMT vs NPPV	Meeting criteria for ETI during 10 h follow-up; in-hospital mortality; AMI incidence	Adequate	Yes	No	Yes	Uncertain	Yes	Adequate
Levitt *et al*. 2001 [14]	USA: ED	38	SMT vs NPPV	ETI decided by attending physician during 24 h follow-up; in-hospital mortality; AMI incidence.	Adequate	Yes	NR	Uncertain	Uncertain	Yes	Uncertain
Nava *et al*. 2003 [19]	Italy: ED	130	SMT vs NPPV	Meeting criteria for ETI during 24 h follow-up; in-hospital mortality; AMI incidence	Adequate	Yes	NR	Yes	Adequate	Yes	Adequate
Mehta *et al*. 1997 [25]	USA: ED	27	CPAP vs NPPV	ETI decided by attending physician during 24 h follow-up; in-hospital mortality; AMI incidence	Adequate	Yes	Yes^h^	Yes	Adequate	Yes	Uncertain
Martin-Bermudez *et al*. 2002 [17]	Spain: ED	80	CPAP vs NPPV	Meeting criteria for ETI during 24 h follow-up; in-hospital mortality; AMI incidence	Uncertain	Yes	NR	Uncertain	Adequate	Yes	Uncertain
Bellone *et al*. 2004 [20]	Italy: ED	46	CPAP vs NPPV	Meeting criteria for ETI during 36 h follow-up; in-hospital mortality; AMI incidence	Adequate	Yes	No	Yes	Adequate	Yes	Adequate
Bellone *et al*. 2005 [24]	Italy: ED	36	CPAP vs NPPV	Meeting criteria for ETI during 36 h follow-up; in-hospital mortality	Adequate	Yes	No	Yes	Adequate	Yes	Adequate
Park *et al*. 2001 [15]	Brazil: ED	26	SMT vs CPAP vs NPPV	ETI decided by attending physician during 1 h follow-up; in-hospital mortality; AMI incidence	Uncertain	Yes	NR	Yes	Uncertain	Yes	Inadequate
Park *et al*. 2004 [23]	Brazil: ED	80	SMT vs CPAP vs NPPV	ETI decided by attending physician during 24 h follow-up; in-hospital mortality; AMI incidence	Adequate	Yes	NR	Yes	Adequate	Yes	Uncertain
Crane *et al*. 2004 [21]	UK: ED	60	SMT vs CPAP vs NPPV	Meeting criteria for ETI during 2 h follow-up; in-hospital mortality; AMI incidence	Adequate	Yes	No	Yes	Adequate	Yes	Adequate

^a^Classified as: adequate, inadequate or uncertain. ^b^Classified as: yes, if inclusion and exclusion criteria for participants are adequately reported; no, if selection criteria are not reported. ^c^Classified as: yes, for articles that implemented blinding at any level; no, for articles reporting not being able to implement blinding of interventions at any level; not reported (NR), for articles that do not make any mention to blinding. ^d^Classified as: yes, if there was an attempt to standardize treatment and care besides the assigned interventions; no, if no attempt to standardize was applied; uncertain, if it was not clearly reported. ^e^Classified as: adequate; inadequate; uncertain. ^f^Classified as: yes; no; not reported (NR). ^g^Classified as: adequate if objective criteria for endotracheal intubation were defined; inadequate if the criteria were not defined; and uncertain if criteria application was unclear (for example, depending on attending physician). ^h^In this study physicians, nurses and patients were blinded by covering the control panel on the device. AMI, acute myocardial infarction; CPAP, continuous positive airway pressure; CU, coronary unit; ED, emergency department; ETI, endotracheal intubation; HDU, high dependency unit; ICU, intensive care unit; NPPV, non-invasive pressure ventilation; SMT, standard medical therapy.

**Table 3:** Specific quality criteria of included randomized trials

Reference	Inclusion criteria^a^	Exclusion criteria	Baseline co-morbidity: AMI, COPD^b^	Intervention in experimental group CPAP	Intervention in experimental group NPPV	Intervention in control group SMT^c^	Objective criteria for endotracheal intubation^d^
Rasanen *et al*. 1985 [62]	Clinical criteria of APE; RR >25/min; PaO_2_/FiO2 <200	COPD; unresponsive; unable to maintain patent airway; lung infection; pulmonary embolism	AMI: control 10/20; CPAP 9/20 COPD: none	CPAP 10 cmH_2_O face mask plus medical therapy	-	SMT	Adequate Criteria for ETI: PaO_2_<50 mmHg; PaCO_2 _> 55 mmHg; RR >35/min; unresponsiveness; airway obstruction
Bersten *et al*. 1991 [63]	Clinical criteria of APE; PaO_2 _< 70 mmHg; PaCO_2 _> 45 mmHg when O_2 _8 l/min	AMI and shock; SBP <90 mmHg; stenotic VHD; COPD and CO_2 _retention	AMI: control 4/20; CPAP 3/19 COPD: none	CPAP 10 cmH_2_O face mask plus medical therapy	-	SMT	Adequate Criteria for ETI: clinical deterioration; PaO_2 _< 70 mmHg with O_2 _100%; PaCO_2 _> 55 mmHg
Lin *et al*. 1995 [57]	Clinical criteria of APE; PaO_2_/FIO_2 _= 200–400; P [A-a] O_2 _> 250 mmHg	Unresponsive; unable to maintain patent airway; shock; septal rupture; stenotic VHD; COPD and CO_2 _retention	AMI: control 11/50; CPAP 10/50 COPD: none	CPAP face mask titrated up – 2.5, 5, 7.5, 10 and 12.5 cmH_2_O plus medical therapy	-	SMT (plus dopamine)	Adequate Criteria for ETI: cardiac resuscitation or clinical deterioration and two of the following – PaCO_2 _> 55 mmHg, PaO_2_/FiO_2 _< 200 mmHg, RR >35
Takeda *et al*. 1997 [11]	Clinical criteria of APE; respiratory distress; PaO_2 _< 80 mmHg while receiving ≥50% O_2_	Not reported	AMI: CPAP 5/15; Control 6/15 COPD: none	CPAP 4–10 cmH_2_O nasal mask plus medical therapy	-	SMT (plus dopamine, dobutamine, norepinephrine and digitalis)	Adequate Criteria for ETI: clinical deterioration and PaO_2_/FiO_2 _<100 mmHg (with FiO_2 _≥70%), PaCO_2 _>55 mmHg
Takeda *et al*. 1998 [12]	Clinical criteria of APE; PaO_2 _< 80 mmHg	Shock; septal or ventricular rupture	All 22 patients with AMI admitted to the coronary unit	CPAP 4–10 cmH_2_O nasal mask plus medical therapy	-	SMT (plus dopamine, dobutamine, norepinephrine)	Adequate Criteria for ETI: clinical deterioration and PaO_2_/FiO_2 _<100 mmHg (with FiO_2 _≥70%) PaCO_2 _>55 mmHg
Kelly *et al*. 2002 [16]	Clinical criteria of APE; RR > 20/min	Pneumonia; pneumothorax; pre-hospital treatment with interventions other than oxygen, diuretics or opiates	AMI: not reported COPD: not reported	CPAP 7.5 cmH_2_O face mask plus medical therapy	-	SMT	Inadequate Criteria for treatment failure: need for intubation (no defined criteria), hypoxemia or hypercapnia and respiratory distress
L'Her, *et al*. 2004 [22]	Clinical criteria of APE Age >75 years; PaO_2_/FiO_2 _<300 mmHg, RR >25/min	GCS <7; Sat O_2 _<85%; SBP <90 mmHg); chronic respiratory insufficiency	AMI: not reported (acute ischemic heart disease: control 6/46; CPAP 7/43) COPD: none	Face mask CPAP 7.5 cmH_2_O plus medical therapy	-	SMT	Adequate Serious complications considered as death or need for ETI within 48 h. Criteria for ETI: cardiac or respiratory arrest; SBP <80 mmHg; progressive hypoxemia (Sat O_2 _<92%); coma or seizures; agitation
Masip *et al*. 2000 [13]	Clinical criteria of APE	AMI; pneumonia; SBP <90 mmHg; CRF; immediate intubation; neurological deterioration	AMI: control 6/18; NPPV 5/19 COPD: control 7/18; NPPV 3/19	-	NPPV face mask, PEEP 5 cmH_2_O, plus medical therapy PSV 15.2 ± 2.4 cmH_2_O	SMT	Adequate Criteria for ETI: cardiac or respiratory arrest, hypoxemia (Sat O_2 _<80%) and muscles fatigue
Levitt *et al*. 2001 [14]	Clinical criteria of APE; RR >30/min	Immediate need for intubation; radiograph not compatible with APE	AMI: none COPD: not reported	-	NPPV S/T mode, face or nasal mask, initial IPAP of 8 and EPAP of 3 cmH_2_O, pressure support of 5 cmH_2_O plus medical therapy PSV 5.0 cmH_2_O	SMT	Uncertain Decision by attending physician based on the following criteria: respiratory distress, deterioration in mental status or vital signs, PaO_2 _<60 mmHg, PaCO_2 _>50 mmHg
Nava *et al*. 2003 [19]	Clinical criteria of APE; PaO_2_/FiO_2 _< 250; RR >30/min	AMI needing thrombolysis; immediate need for intubation; Kelly score >3; shock; arrhythmias; SpO_2_<80%; severe CRF; pneumothorax	AMI: control 11/65; NPPV 11/65 COPD: control 26/65; NPPV 27/65	-	NPPV S mode face mask IPAP 14.5 ± 21.1 cmH_2_O, EPAP: 6.1 ± 3.2 cmH_2_O plus medical therapy PSV 8.4 cmH2O	SMT	Adequate Sat O_2 _<85% with FiO_2 _100%, cardiac or respiratory arrest, inability to tolerate mask, PaCO_2 _>50 mmHg, signs of pump exhaustion, SBP <90 mmHg, AMI, massive GI bleeding
Mehta *et al*. 1997 [25]	Clinical criteria of APE; RR >30/min; tachycardia >100 bpm; without pulmonary aspiration or infection	Immediate need for intubation; respiratory or cardiac arrest; arrhythmias; SBP <90 mmHg; unresponsive; agitated; condition precluding use of face mask	AMI: CPAP 1/13; NPPV 1/14 Chest pain: CPAP 4/13; NPPV 10/14; COPD: not reported	CPAP 10 cmH_2_O nose/face mask plus medical therapy	NPPV S/T mode, nasal/face mask, IPAP 15 cmH2O, EPAP 5 cmH2O, plus medical therapy PSV 10.0 cmH_2_O	-	Uncertain Decision by attending physician based on the following criteria: severe respiratory distress, inability to tolerate mask, unstable vital signs, PaO_2 _<60 mmHg or increase PaCO_2 _>5 mmHg
Martin-Bermudez, *et al*. 2002 [17]	Clinical criteria of APE; RR >25/min; Sat O_2 _<90%	Not reported	AMI: not reported COPD: not reported	Face mask CPAP plus medical therapy	Face mask NPPV plus medical therapy PSV uncertain	-	Uncertain
Bellone *et al*. 2004 [20]	Clinical criteria of APE; Sat O_2 _<90%; RR >30/min	Acute coronary syndrome; immediate need for intubation; respiratory or cardiac arrest; SBP <90 mmHg; unresponsive, agitated or unable to cooperate; condition precluding use of face mask	AMI: none COPD: CPAP 8/22; NPPV 6/24	Face mask CPAP 10 cmH_2_O plus medical therapy	Face mask NPPV initially IPAP 15 cmH_2_O and EPAP 5 cmH_2_O, with adjustments as needed to obtain tidal volume >400 ml plus medical therapy PSV 10.0 cmH_2_O	-	Adequate Respiratory arrest; loss of consciousness; agitation; heart rate <50/min, SBP <70 mmHg
Bellone *et al*. 2005 [24]	Clinical criteria of APE; PaCO_2 _> 45 mmHg; Sat O_2 _<90%; RR >30/min	COPD; PaCO2 <45 mmHg; immediate need for intubation; respiratory or cardiac arrest; SBP <90 mmHg; CRF; agitated; condition precluding use of face mask; enrolled in other study	AMI: CPAP 0/18; NPPV 2/18 COPD: none	Face mask CPAP 10 cmH_2_O plus medical therapy	Face mask NPPV initially IPAP 15 cmH_2_O, EPAP 5 cmH_2_O, adjustments to obtain tidal volume >400 ml plus medical therapy PSV 10.0 cmH_2_O	-	Adequate Respiratory arrest; loss of consciousness; agitation; heart rate <50/min, SBP <70 mmHg
Park *et al*. 2001 [15]	Clinical criteria of APE; RR >25/min	COPD; SBP <90 mmHg; arrhythmias; bradypnea; unresponsive, agitated or unable to cooperate; vomiting; digestive hemorrhage; facial deformities	AMI: control 2/10; CPAP 1/9; NPPV 1/7 COPD: none	Face mask CPAP mean 7.5 cmH_2_O, initially 5, increased by 2.5, maximum 12.5 cmH_2_O, plus medical therapy	NPPV S/T mode nasal mask, IPAP 12 cmH_2_O, EPAP 4 cmH_2_O, plus medical therapy PSV 8.0 cmH_2_O	SMT	Inadequate Decision made by the attending physician based on clinical and laboratory findings
Park *et al*. 2004 [23]	Clinical criteria of APE; RR >25/min	AMI; COPD; pulmonary embolism; pneumonia; pneumothorax; SBP <90 mmHg; vomiting	AMI: control 3/26; CPAP 1/27; NPPV 1/27 COPD: none	Face mask CPAP initially 11 ± 2 cmH_2_O plus medical therapy	Face mask NPPV, IPAP 17 ± 2 cmH_2_O, EPAP 11 ± 2 cmH_2_O, plus medical therapy PSV 6.0 cmH_2_O	SMT	Uncertain Decision made by the attending physician based on the following criteria: GCS <13, respiratory distress, PaO_2 _<60 mmHg, Sat O_2 _<90%, increase PaCO_2 _>5 mmHg
Crane *et al*. 2004 [21]	Clinical criteria of APE; RR >23/min; pH <7.35	SBP <90 mmHg; temperature >38°C; AMI with thrombolysis; dialysis for CRF; impaired consciousness; dementia	AMI: none COPD: control 6/20; CPAP 3/20; NPPV 7/20	Face mask CPAP 10 cmH O plus medical therapy	Face mask NPPV IPAP 15 cmH_2_O, EPAP 5 cmH_2_O plus medical therapy PSV 10.0 cmH_2_O	SMT	Adequate RR >40 or <10 and reduced consciousness; falling pH (<7.2)

^a^Clinical criteria of APE: existence of dyspnea of sudden onset, bilateral pulmonary infiltrates on chest radiograph and a compatible physical examination (bilateral crackles on pulmonary auscultation, elevated jugular venous pressure, third heart sound on cardiac auscultation). ^b^Data on baseline frequency of acute myocardial infarction (AMI) and chronic obstructive pulmonary disease (COPD) are presented as number of patients with co-morbidity/total number of patients in the assigned group. ^c^Standard medical therapy was defined as: O_2 _by face mask, nitro-glycerin, nitroprusside, furosemide and morphine. Other interventions described in managing these patients will be specifically indicated. ^d^Classified as: adequate if objective criteria for endotracheal intubation were defined; inadequate if the criteria were not defined; and uncertain if criteria application was unclear (for example, depending on attending physician). APE, acute pulmonary edema; bpm, beats per minute; CPAP, continuous positive airway pressure; CRF, chronic renal failure; DBP, diastolic blood pressure; EPAP, expiratory positive airway pressure; ETI, endotracheal intubation; FiO_2_, O_2 _inspired fraction; GI, Gastrointestinal; GCS, Glasgow coma scale; IPAP, inspiratory positive airway pressure; NPPV, non-invasive pressure ventilation; P [A-a], arterial/alveolar partial pressure differential; PaCO_2_, CO_2 _partial pressure; PaO_2_, O_2 _partial pressure; PEEP, positive end expiratory pressure; PSV, pressure support ventilation; RR, respiratory rate; Sat O_2_, O_2 _saturation; SBP, systolic blood pressure; S mode, spontaneous mode; SMT, standard medical therapy; SpO2, pulse oximetry oxygen saturation; S/T mode, spontaneous/timed mode; VHD, valvular heart disease.

## References

[B1] Jessup M, Brozena S (2003). Heart Failure. N Engl J Med.

[B2] American Heart Association (2004). 2004 Heart and Stroke statistical update.

[B3] Fonarow G, ADHERE scientific advisory committee (2003). The acute decompensated heart failure national registry (ADHERE): opportunities to improve care of patients hospitalized with acute decompensated heart failure. Rev Cardiovasc Med.

[B4] Fedullo AJ, Swinburne AJ, Wahl GW, Bixby K (1991). Acute cardiogenic pulmonary edema treated with mechanical ventilation. Factors determining in-hospital mortality. Chest.

[B5] Antonelli M, Pennisi MA, Montini L (2005). Clinical review: Noninvasive ventilation in the clinical setting-experience from the past 10 years. Crit Care.

[B6] Girou E, Brun-Buisson C, Taille S, Lemaire F, Brochard L (2003). Secular trends in nosocomial infections and mortality associated with noninvasive ventilation in patients with exacerbation of COPD and pulmonary edema. JAMA.

[B7] Antonelli M, Conti G, Rocco M, Bufi M, De Blasi RA, Vivino G, Gasparetto A, Meduri GU (1998). A comparison of noninvasive positive-pressure ventilation and conventional mechanical ventilation in patients with acute respiratory failure. N Engl J Med.

[B8] Vaisanen IT, Rasanen J (1987). Continuous positive airway pressure and supplemental oxygen in the treatment of cardiogenic pulmonary edema. Chest.

[B9] Hoffmann B, Welte T (1999). The use of noninvasive pressure support ventilation for severe respiratory insufficiency due to pulmonary oedema. Intensive Care Med.

[B10] Pang D, Keenan SP, Cook DJ, Sibbald WJ (1998). The effect of positive pressure airway support on mortality and the need for intubation in cardiogenic pulmonary edema: a systematic review. Chest.

[B11] Takeda S, Takano T, Ogawa R (1997). The effect of nasal continuous positive airway pressure on plasma endothelin-1 concentrations in patients with severe cardiogenic pulmonary edema. Anesth Analg.

[B12] Takeda S, Nejima J, Takano T, Nakanishi K, Takayama M, Sakamoto A, Ogawa R (1998). Effect of nasal continuous positive airway pressure on pulmonary edema complicating acute myocardial infarction. Jpn Circ J.

[B13] Masip J, Betbese AJ, Paez J, Vecilla F, Canizares R, Padro J, Paz MA, de Otero J, Ballus J (2000). Non-invasive pressure support ventilation versus conventional oxygen therapy in acute cardiogenic pulmonary oedema: a randomised trial. Lancet.

[B14] Levitt MA (2001). A prospective, randomized trial of BiPAP in severe acute congestive heart failure. J Emerg Med.

[B15] Park M, Lorenzi-Filho G, MI Feltrim, PR Viecili, MC Sangean, M Volpe, Leite PF, Mansur AJ (2001). Oxygen therapy, continuous positive airway pressure, or noninvasive bilevel positive pressure ventilation in the treatment of acute cardiogenic pulmonary edema. Arq Bras Cardiol.

[B16] Kelly CA, Newby DE, McDonagh TA, Mackay TW, Barr J, Boon NA, Dargie HJ, Douglas NJ (2002). Randomised controlled trial of continuous positive airway pressure and standard oxygen therapy in acute pulmonary oedema; effects on plasma brain natriuretic peptide concentrations. Eur Heart J.

[B17] Martin-Bermudez RJ, Rodriguez-Portal JA, Garcia-Garmendia JL, Garcia-Diaz E, Montano-Diaz M, Soto-Espinosa B, Murillo-Cabezas F, Muniz-Grijalvo O (2002). Non-invasive ventilation in cardiogenic pulmonary edema. Preliminary results of a randomized trial. Intensive Care Med.

[B18] Cross AM, Cameron P, Kierce M, Ragg M, Kelly AM (2003). Non-invasive ventilation in acute respiratory failure: a randomised comparison of continuous positive airway pressure and bi-level positive airway pressure. Emerg Med J.

[B19] Nava S, Carbone G, DiBattista N, Bellone A, Baiardi P, Cosentini R, Marenco M, Giostra F, Borasi G, Groff P (2003). Noninvasive ventilation in cardiogenic pulmonary edema: a multicenter randomized trial. Am J Respir Crit Care Med.

[B20] Bellone A, Monari A, Cortellaro F, Vettorello M, Arlati S, Coen D (2004). Myocardial infarction rate in acute pulmonary edema: noninvasive pressure support ventilation versus continuous positive airway pressure. Crit Care Med.

[B21] Crane SD, Elliott MW, Gilligan P, Richards K, Gray AJ (2004). Randomised controlled comparison of continuous positive airways pressure, bilevel non-invasive ventilation, and standard treatment in emergency department patients with acute cardiogenic pulmonary oedema. Emerg Med J.

[B22] L'Her E, Duquesne F, Girou E, de Rosiere XD, Le Conte P, Renault S, Allamy JP, Boles JM (2004). Noninvasive continuous positive airway pressure in elderly cardiogenic pulmonary edema patients. Intensive Care Med.

[B23] Park M, Sangean MC, Volpe Mde S, Feltrim MI, Nozawa E, Leite PF, Passos Amato MB, Lorenzi-Filho G (2004). Randomized, prospective trial of oxygen, continuous positive airway pressure, and bilevel positive airway pressure by face mask in acute cardiogenic pulmonary edema. Crit Care Med.

[B24] Bellone A, Vettorello M, Monari A, Cortellaro F, Coen D (2005). Noninvasive pressure support ventilation vs. continuous positive airway pressure in acute hypercapnic pulmonary edema. Intensive Care Med.

[B25] Mehta S, Jay GD, Woolard RH, Hipona RA, Connolly EM, Cimini DM, Drinkwine JH, Hill NS (1997). Randomized, prospective trial of bilevel versus continuous positive airway pressure in acute pulmonary edema. Crit Care Med.

[B26] Masip J, Paez J, Betbese AJ, Vecilla F (2004). Noninvasive ventilation for pulmonary edema in the emergency room. Am J Respir Crit Care Med.

[B27] Masip J, Roque M, Sanchez B, Fernandez R, Subirana M, Exposito JA (2005). Noninvasive Ventilation in Acute Cardiogenic Pulmonary Edema-Systematic Review and Meta-analysis. JAMA.

[B28] Moher D, Cook DJ, Eastwood S, Olkin I, Rennie D, Stroup DF (1999). Improving the quality of reports of meta-analysis of randomised controlled trials: The QUOROM statement. Lancet.

[B29] Ferrer M, Esquinas A, Leon M, Gonzalez G, Alarcon A, Torres A (2003). Noninvasive ventilation in severe hypoxemic respiratory failure: a randomized trial. Am J Respir Crit Care Med.

[B30] Delclaux C, L'Her E, Alberti C, Mancebo J, Abroug F, Conti G, Guerin C, S F, Lefort Y, Antonelli M (2000). Treatment of acute hypoxemic nonhypercapnic respiratory insufficiency with continuous airway pressure delivered by a face mask: a randomized controlled trial. JAMA.

[B31] Antonelli M, Conti G, Bufi M, Costa MG, Lappa A, Rocco M, Gasparetto A, Meduri GU (2000). Noninvasive ventilation for treatment of acute respiratory failure in patients undergoing solid organ transplantation: a randomized trial. JAMA.

[B32] Wood KA, Lewis L, Von Harz B, Kollef MH (1998). The use of noninvasive positive pressure ventilation in the emergency department: results of a randomized clinical trial. Chest.

[B33] Wysocki M, Tric L, Wolff MA, Millet H, Herman B (1995). Noninvasive pressure support ventilation in patients with acute respiratory failure. A randomized comparison with conventional therapy. Chest.

[B34] Schechtman E (2002). Odds ratio, relative risk, absolute risk reduction, and the number needed to treat – which of these should we use?. Value Health.

[B35] Walter SD (2000). Choice of effect measure for epidemiological data. J Clin Epidemiol.

[B36] Begg CB, Mazumdar M (1994). Operating characteristics of a rank correlation test for publication bias. Biometrics.

[B37] Review Manager (RevMan) [Computer program] (2002). Version 4.2 for Windows.

[B38] RevMan Analyses [Computer program] (2002). Version 1.0 for Windows.

[B39] Craven RA, Singletary N, Bosken L, Sewell E, Payne M, Lipsey R (2000). Use of bilevel positive airway pressure in out-of-hospital patients. Acad Emerg Med.

[B40] Sarullo FM, D'Alfonso G, Brusca I, De Michele P, Taormina A, Di Pasquale P, Castello A (2004). Efficacy and safety of non-invasive positive pressure ventilation therapy in acute pulmonary edema. Monaldi Arch Chest Dis.

[B41] Giacomini M, Iapichino G, Cigada M, Minuto A, Facchini R, Noto A, Assi E (2003). Short-term noninvasive pressure support ventilation prevents ICU admittance in patients with acute cardiogenic pulmonary edema. Chest.

[B42] Valipour A, Cozzarini W, Burghuber OC (2004). Non-invasive pressure support ventilation in patients with respiratory failure due to severe acute cardiogenic pulmonary edema. Respiration.

[B43] Rusterholtz T, Kempf J, Berton C, Gayol S, Tournoud C, Zaehringer M, Jaeger A, Sauder P (1999). Noninvasive pressure support ventilation (NIPSV) with face mask in patients with acute cardiogenic pulmonary edema (ACPE). Intensive Care Med.

[B44] Wigder HN, Hoffmann P, Mazzolini D, Stone A, Scholly S, Clark J (2001). Pressure support noninvasive positive pressure ventilation treatment of acute cardiogenic pulmonary edema. Am J Emerg Med.

[B45] Baratz DM, Westbrook PR, Shah PK, Mohsenifar Z (1992). Effect of nasal continuous positive airway pressure on cardiac output and oxygen delivery in patients with congestive heart failure. Chest.

[B46] Lenique F, Habis M, Lofaso F, Dubois-Rande JL, Harf A, Brochard L (1997). Ventilatory and hemodynamic effects of continuous positive airway pressure in left heart failure. Am J Respir Crit Care Med.

[B47] Kallio T, Kuisma M, Alaspaa A, Rosenberg PH (2003). The use of prehospital continuous positive airway pressure treatment in presumed acute severe pulmonary edema. Prehosp Emerg Care.

[B48] Thys F, Roeseler J, Reynaert M, Liistro G, Rodenstein DO (2002). Noninvasive ventilation for acute respiratory failure: a prospective randomised placebo-controlled trial. Eur Respir J.

[B49] Yosefy C, Hay E, Ben-Barak A, Derazon H, Magen E, Reisin L, Scharf S (2003). BiPAP ventilation as assistance for patients presenting with respiratory distress in the department of emergency medicine. Am J Respir Med.

[B50] Mollica C, Brunetti G, Buscajoni M, Cecchini L, Maialetti E, Marazzi M, Principe R, Sabato R, Antonini VE (2001). Non-invasive pressure support ventilation in acute hypoxemic (non hypercapnic) respiratory failure. Observations in Respiratory Intermediate Intensive Care Unit. Minerva Anestesiol.

[B51] L'Her E, Moriconi M, Texier F, Bouquin V, Kaba L, Renault A, Garo B, Boles JM (1998). Non-invasive continuous positive airway pressure in acute hypoxaemic respiratory failure-experience of an emergency department. Eur J Emerg Med.

[B52] Bollaert PE, Sauder P, Girard F, Rusterholtz T, Feissel M, Harlay ML, Zaehringer M, Dusang B (2002). Continuous positive airway pressure (CPAP) vs. Proportional Assist Ventilation (PAV) for noninvasive ventilation in cardiogenic pulmonary edema: a randomized study. Am J Respir Crit Care Med.

[B53] Moritz F, Benichou J, Vanheste M, Richard JC, Line S, Hellot MF, Bonmarchand G, Muller JM (2003). Boussignac continuous positive airway pressure device in the emergency care of acute cardiogenic pulmonary oedema: a randomized pilot study. Eur J Emerg Med.

[B54] Chadda K, Annane D, Hart N, Gajdos P, Raphael JC, Lofaso F (2002). Cardiac and respiratory effects of continuous positive airway pressure and noninvasive ventilation in acute cardiac pulmonary edema. Crit Care Med.

[B55] M Lin, HT Chiang (1991). The efficacy of early continuous positive airway pressure therapy in patients with acute cardiogenic pulmonary edema. J Formos Med Assoc.

[B56] Crane SD, Richards K, Gilligan P, Gray A, Elliott MW (2004). Randomised controlled trial of non-invasive ventilation in acute cardiogenic pulmonary oedema (3PO study). Emerg Med J.

[B57] Lin M, Yang YF, Chiang HT, Chang MS, Chiang BN, Cheitlin MD (1995). Reappraisal of continuous positive airway pressure therapy in acute cardiogenic pulmonary edema. Short-term results and long-term follow-up. Chest.

[B58] Hao CX, Luo XR, Liu YM (2002). Treatment of severe cardiogenic pulmonary edema with continuous positive airway pressure by basal face mask. Acta Academiae Medicinae Jiangxi.

[B59] Sharon A, Shpirer I, Kaluski E, Moshkovitz Y, Milovanov O, Polak R, Blatt A, A Simovitz, Shaham O, Faigenberg Z (2000). High-dose intravenous isosorbide-dinitrate is safer and better than Bi-PAP ventilation combined with conventional treatment for severe pulmonary edema. J Am Coll Cardiol.

[B60] Brochard L (2002). Noninvasive ventilation for Acute Respiratory Failure. JAMA.

[B61] Liesching TN, Cromier K, Nelson D, Short K, Sucov A, Hill NS (2003). Bilevel Noninvasive Ventilation vs Continuous Positive Airway Pressure to treat Acute Pulmonary Edema. Am J Respir Crit Care Med.

[B62] Rasanen J, Heikkila J, Downs J, Nikki P, Vaisanen I, Viitanen A (1985). Continuous positive airway pressure by face mask in acute cardiogenic pulmonary edema. Am J Cardiol.

[B63] Bersten AD, Holt AW, Vedig AE, Skowronski GA, Baggoley CJ (1991). Treatment of severe cardiogenic pulmonary edema with continuous positive airway pressure delivered by face mask. N Engl J Med.

[B64] Adnet F, Le Toumelin P, Leberre A, Minadeo J, Lapostolle F, Plaisance P, Cupa M (2001). In-hospital and long-term prognosis of elderly patients requiring endotracheal intubation for life-threatening presentation of cardiogenic pulmonary edema. Crit Care Med.

[B65] Poulton PE (1936). Left-sided heart failure with pulmonary edema:its treatment with the"pulmonary plus pressure machine". Lancet.

[B66] Holt AW, Bersten AD, Fuller S, Piper RK, Worthley LI, Vedig AE (1994). Intensive care costing methodology: cost benefit analysis of mask continuous positive airway pressure for severe cardiogenic pulmonary oedema. Anaesth Intensive Care.

[B67] Lightowler JV, Wedzicha JA, Elliott MW, Ram FS (2003). Non-invasive positive pressure ventilation to treat respiratory failure resulting from exacerbations of chronic obstructive pulmonary disease: Cochrane systematic review and meta-analysis. BMJ.

[B68] Masip J, Paez J, Merino M, Parejo S, Vecilla F, Riera C, Rios A, Sabater J, Ballus J, Padro J (2003). Risk factors for intubation as a guide for noninvasive ventilation in patients with severe acute cardiogenic pulmonary edema. Intensive Care Med.

[B69] Kwok H, McCormack J, Cece R, Houtchens J, Hill NS (2003). Controlled trial of oronasal versus nasal mask ventilation in the treatment of acute respiratory failure. Crit Care Med.

[B70] Gay PC, Hess DR, Hill NS (2001). Noninvasive proportional assist ventilation for acute respiratory insufficiency. Comparison with pressure support ventilation. Am J Respir Crit Care Med.

[B71] Nieminen MS, Bohm M, Cowie MR, Drexler H, Filippatos GS, Jondeau G, Hasin Y, Lopez-Sendon J, Mebazaa A (2005). Executive summary of the guidelines on the diagnosis and treatment of acute heart failure: the Task Force on Acute Heart Failure of the European Society of Cardiology. Eur Heart J.

